# Surgical Treatment of Pancreatic Ductal Adenocarcinoma

**DOI:** 10.3390/cancers13164015

**Published:** 2021-08-10

**Authors:** Sohei Satoi

**Affiliations:** Department of Surgery, Kansai Medical University, Hirakata 573-1010, Japan; satoi@hirakata.kmu.ac.jp

This special issue, “Surgical Treatment of Pancreatic Ductal Adenocarcinoma” contains 13 articles (five original articles, five reviews, and three systematic reviews/meta-analyses) authored by international leaders and surgeons who treat patients with pancreatic ductal adenocarcinoma (PDAC). 

Oncological pancreatic surgery requires a deep knowledge of multimodal treatment, accurate preoperative recognition of tumor extension—especially to adjacent major vessels—high-quality technical skills for margin-negative resection, and well-established perioperative management for the reduction of morbidity and mortality. In the modern era, it involves a two-sided advancement toward extended pancreatectomy, such as portal vein or major arterial resection for locally advanced PDAC, as well as minimally invasive surgery for resectable PDAC.

Surgical resection has provided the only chance for a cure in patients with PDAC, but the 5 year survival rate is still low (approximately 20%) in patients with margin-negative resection. The implementation of adjuvant chemotherapy or neoadjuvant therapy has dramatically increased the long term survival of patients with resectable, borderline resectable, and even unresectable PDAC. Margin-negative surgical resection still plays a pivotal role in multimodal treatment in patients with PDAC. Therefore, a growing amount of interest has focused on optimization of the perioperative therapeutic strategy, including multimodal treatment regimens, the introduction of extended pancreatectomy, and advanced perioperative management. Moreover, the introduction of minimally invasive surgery, such as laparoscopic and robotic pancreatic surgery, has been applied worldwide.

This special issue highlights the role of surgical resection in patients with PDAC to advance our understanding of the surgical treatment of PDAC.

With regard to the influence of tumor location on prognosis, among 2483 patients with all types of PDAC, long term survival was significantly better for patients with pancreatic head/uncinate PDAC than with body/tail PDAC, regardless of resectability [1]. Among patients who underwent curative resection, those with head/uncinate cancers had a higher number of T1/T2 tumors, but worse outcomes. Multivariate analysis identified tumor factors, preoperative CA 19-9 level, margin status, and adjuvant therapy, but not tumor location as independent prognostic factors. Margin-negative resection during multimodal treatment is mandatory for long term survival in patients with PDAC.

What can we do to optimize the rate of margin-negative resection? According to the “appropriate dissection range” identified with simulated use of high-quality computed tomography preoperatively, surgeons should carry out “dissection to achieve margin-negative resection”, identifying anatomical structures, such as layers, arteries, and veins, as anatomical landmarks to determine the dissection region intraoperatively [2]. 

Neoadjuvant therapy has been implemented recently to achieve a high proportion of margin-negative resection and negative lymph node metastasis through anatomical and biological shrinkage of borderline resectable tumors. Neoadjuvant therapy followed by surgery, rather than upfront surgery, has been reported to offer clinical benefits to patients with borderline resectable PDAC [3]. Moreover, it is suggested that nutritional management during neoadjuvant therapy may lead to a better prognosis. 

Given the multimodal approach with new chemotherapy regimens, such as fluorouracil plus leucovorin, irinotecan, oxaliplatin (FOLFIRINOX), or gemcitabine plus nab-paclitaxel, substantial progress has been made in surgical techniques to address advanced resections [4]. Margin-negative resection in patients with locally advanced PDAC usually requires portal vein or major arterial resection and reconstruction. These mainly vessel-oriented technical approaches of pancreatic head resection allow the removal of all putative tumor-infiltrated soft tissue with the utmost aim for an improved R0 resection rate [4].

Aggressive pancreatectomy, such as total pancreatectomy or combined arterial resection, for achieving margin-negative resection has become justified by the principle of total neoadjuvant therapy in recent decades [5]. Further technical standardization and an optimal neoadjuvant strategy are mandatory for the global adoption of aggressive pancreatectomy.

Recently, surgical resection in PDAC has been extended to patients with unresectable PDAC. Additional surgery during multimodal treatment is defined as “conversion surgery” in patients with unresectable PDAC (metastatic and locally advanced disease) which comprises 70–80% of the PDAC population. Although surgical resectability was less than 10% in 398 patients [6] and 469 patients [7] with unresectable PDAC, including metastatic disease, the median survival time after initial treatment was 37 months and 73.7 months in patients who underwent conversion surgery, respectively. The number of candidates for conversion surgery is now increasing with the introduction of modern chemotherapy regimens; however, the actual clinical benefits of resection have not yet been fully investigated. Prospective studies will be needed to explore the clinical benefit of conversion surgery. 

A high rate of recurrence, even after margin-negative resection, has been reported in patients with PDAC. Recurrent PDAC, mainly containing liver or peritoneal metastasis, and local recurrence is commonly treated with systemic chemotherapy or best supportive care. The clinical role of surgical resection for patients with isolated local recurrent PDAC after initial pancreatectomy is still under investigation. Although there is a possibility of selection bias, meta-analysis revealed that surgical resection in selected patients with recurrent pancreatic cancer was safe and feasible and might offer a survival advantage [8]. This meta-analysis also suggested that surgery should be considered part of the multimodal management of relapsing pancreatic cancer, and a multidisciplinary approach is essential to choose the most appropriate treatment [8]. Thus, PDAC surgery in the modern era frequently requires extended pancreatectomy; therefore, appropriate patient selection is mandatory. The development of precise biological and anatomical assessments will be urgently needed.

Therefore, novel biomarkers predicting resectability, overall survival, and disease-free survival should be established promptly. Liquid biopsy involving cancer DNA and circulating tumor cells in the blood may be an additional tool for estimating disease course and outcome in patients with PDAC [9]. Clinical application of liquid biopsy may provide a cancer diagnosis at an earlier stage, enable optimal selection of treatment, and inform prediction of prognosis and recurrence.

While extended pancreatectomy has been developed safely and effectively in patients with locally advanced PDAC, minimally invasive pancreatic surgery has also evolved. Laparoscopic and robotic pancreatectomy are considered safe and feasible for experienced surgeons in well-selected patients with PDAC. With the advancement of minimally invasive techniques and experiences, laparoscopic distal pancreatectomy (LDP) and even laparoscopic pancreaticoduodenectomy (LPD) have been implemented successfully for treating PDAC [10]. However, due to a limited volume of evidence, without doubt, there is a strong need for more high-quality trials to confirm the potential advantages of minimally invasive pancreatic surgery [4]. 

Optimizing existing pathways for PDAC treatment so that patients realize the benefits of already proven treatments presents a clear opportunity to improve outcomes in the short term. The narrative review [11] focuses on treatments and interventions where there was a clear evidence base to improve outcomes in pancreatic cancer and where there was evidence of variation and undertreatment. The avoidance of preoperative biliary drainage, treatment of pancreatic exocrine insufficiency, prehabilitation and enhanced recovery after surgery, reduction of perioperative complications, optimization of opportunities for elderly patients to receive therapy, optimization of adjuvant chemotherapy, and regular surveillance after surgery are some of the strategies discussed. Each treatment or pathway change represents an opportunity for marginal gain, and the accumulation of marginal gains can result in a considerable benefit to patients. It is essential that surgeons understand that surgery is just one part of a complex pathway and that they are ideally placed to act as change agents to optimize broader pathway improvements.

Other concerns are risk factors for malignancy, defined as high-grade dysplasia and invasive carcinoma in patients with intraductal papillary mucinous neoplasm (IPMN). One meta-analysis revealed risk factors for malignancy as symptoms, size ≥ 3 cm, cystic wall thickening, mural nodule, main pancreatic duct dilatation, abrupt caliber change in the pancreatic duct, lymphadenopathy, elevated carbohydrate antigen 19-9 level, and elevated carcinoembryonic antigen level [12]. Among the above risk factors, the role of main pancreatic duct (MPD) dilatation is important for establishing a simple surgical indication. However, the degree of ductal dilatation that warrants pancreatectomy is still controversial across the existing guidelines. The other meta-analysis concluded that MPD dilatation was an important predictive factor of IPMN malignancy, and 5 mm was a highly sensitive cutoff for the detection of high-risk pre-cancerous or cancerous lesions in resected patients. The need for pancreatectomy should be thoroughly evaluated in patients with ductal dilatations of ≥5 mm for improving surgical patient selection and reducing overall IPMN malignancy mortality [13].

In conclusion, surgical treatment of PDAC has experienced a paradigm shift, from “the only way for cure” in the last century, to “the essential position during multimodal treatment” in the modern era. Pancreatic surgery for PDAC now has two-sided progress. Extended pancreatectomy with vessel resection and reconstruction has been performed safely and effectively in patients with locally advanced PDAC following multimodal treatment. In contrast, the implementation of minimally invasive surgery is also useful in selected patients with PDAC. The establishment of an appropriate surgical indication for predicting an acceptable prognosis is required in the era of multimodal treatment. Biomarkers that inform a surgical indication may be revealed by liquid biopsy in the near future. Sustainable efforts are warranted to establish a role for surgical treatment during multimodal treatment in patients with PDAC who still have high lethality.
